# First real-world evidence of sparsentan efficacy in patients with IgA nephropathy treated with SGLT2 inhibitors

**DOI:** 10.1093/ckj/sfae394

**Published:** 2024-12-03

**Authors:** Moritz Schanz, Claudia Seikrit, Bernd Hohenstein, Aline Zimmermann, Leonie Kraft, Severin Schricker, Susann Berger, Andrea Schwab, Tina Oberacker, Joerg Latus

**Affiliations:** Department of General Internal Medicine and Nephrology, Robert Bosch Hospital Stuttgart, Stuttgart, Germany; Division of Nephrology and Clinical Immunology, RWTH Aachen University Hospital, Aachen, Germany; Nephrological Center Villingen-Schwenningen, Villingen-Schwenningen, Germany; Dialysis Center of the Arbeitsgemeinschaft Heimdialyse Saar e.V., Homburg, Germany; Department of General Internal Medicine and Nephrology, Robert Bosch Hospital Stuttgart, Stuttgart, Germany; Department of General Internal Medicine and Nephrology, Robert Bosch Hospital Stuttgart, Stuttgart, Germany; Department of General Internal Medicine and Nephrology, Robert Bosch Hospital Stuttgart, Stuttgart, Germany; Department of General Internal Medicine and Nephrology, Robert Bosch Hospital Stuttgart, Stuttgart, Germany; Dr Margarete Fischer-Bosch-Institute of Clinical Pharmacology and University of Tuebingen, Stuttgart, Germany; Department of General Internal Medicine and Nephrology, Robert Bosch Hospital Stuttgart, Stuttgart, Germany

**Keywords:** chronic kidney disease (CKD), dual endothelin angiotensin receptor antagonist (DEARA), IgA nephropathy, sodium-glucose cotransporter-2 (SGLT-2) inhibition, sparsentan

## Abstract

**Background:**

Sparsentan, a dual-acting antagonist for both the angiotensin II receptor type 1 and the endothelin receptor type A, has emerged as a promising therapeutic agent for the treatment of IgA nephropathy (IgAN). Following the publication of the PROTECT trial, sparsentan recently received approval for the treatment of IgAN in Europe. However, it remains uncertain whether an additive effect can be observed in the context of existing treatment with sodium-glucose co-transporter 2 (SGLT2) inhibitors, given that the PROTECT study did not investigate this dual therapy approach.

**Methods:**

A total of 23 patients with IgAN were treated with sparsentan via the Managed Access Programme between December 2023 and August 2024. The patients were stable on maximum tolerated doses of renin–angiotensin system (RAS) and SGLT2 inhibitors, with an estimated glomerular filtration rate (eGFR) >30 mL/min/1.73 m² and a urine protein/creatinine ratio (UPCR) >0.75 g/g.

**Results:**

In the 23 patients, median (IQR) baseline eGFR (CKD-EPI) was 42 mL/min/1.73 m^2^ (32–63) and median baseline UPCR was 1.5 g/g (0.9–1.8). After initiation of sparsentan, UPCR significantly decreased (*P* < 0.0001) to a median of 0.85 g/g (0.42–1.15) in the 2-week follow-up and further declined (*P* = 0.001) to a median of 0.60 g/g (0.32–0.82) after 14 weeks, equivalent to a relative reduction in proteinuria up to 62% (45–74). A similar significant reduction was observed for the urine albumin/creatinine ratio. No drug-related serious adverse events were reported.

**Conclusions:**

In this real-world setting, sparsentan shows a significant impact on proteinuria, leading to a relative reduction of 62% in UPCR after 14 weeks and beyond, even in patients already receiving SGLT2 inhibitors.

## Graphical Abstract

**Graphical Abstract:**
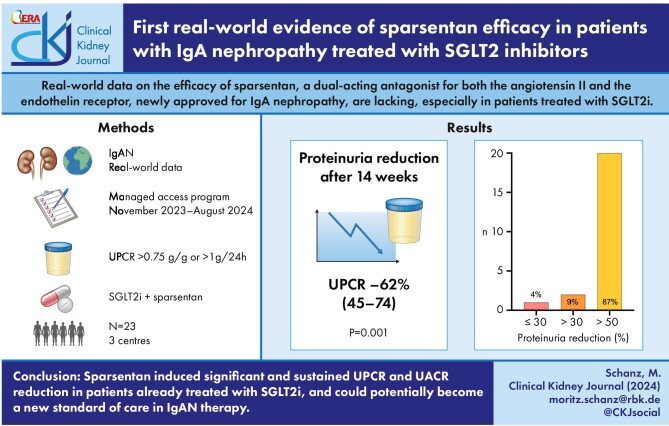

KEY LEARNING POINTS **What was known:**

- Sparsentan, a dual-acting antagonist for both the angiotensin II receptor type 1 and the endothelin receptor type A, has been recently approved for the treatment of IgA nephropathy (IgAN), exhibiting significant efficacy in reducing proteinuria and slowing down IgAN progression.
- Information on effects of sparsentan on top of pre-existing SGLT2 inhibition are lacking.
- Moreover, real-world data on sparsentan efficacy treating IgAN are currently lacking.

**This study adds:**

- Sparsentan demonstrated additional effects on reducing proteinuria in patients who were stable on the maximum tolerated doses of renin–angiotensin system and SGLT2 inhibitors.
- Our data confirm that sparsentan causes a significant and sustained reduction in urine protein/creatinine ratio and urine albumin/creatinine ratio.
- Sparsentan was well tolerated on top of SGLT2 inhibition with no serious adverse events and only a few, mild adverse events.

**Potential impact:**

- Sparsentan shows promising effects, potentially becoming a new standard of care in IgAN therapy.

## INTRODUCTION

IgA nephropathy (IgAN), is a rare but the most common primary glomerular disease worldwide and a leading cause of kidney failure, especially in younger adults. The progression of IgAN leads to advanced CKD and kidney failure in up to 50% of IgAN patients [1]. Therefore, early detection and optimal treatment are of high importance. Proteinuria is an established risk factor for IgAN disease progression and prognosis [2]. In general, reducing proteinuria <1 g/day is considered a surrogate marker for improved kidney outcomes in IgAN and a reasonable treatment target according to the Kidney Disease: Improving Global Outcomes (KDIGO) 2021 guidelines [3]. Nevertheless, data of the German CKD (GCKD) cohort and the UK National Registry of Rare Kidney Diseases (RaDaR) revealed that even ‘low-risk’ patients, with proteinuria [urine protein/creatinine ratio (UPCR)] between 0.1 and 0.6 g/g, face a significant risk of becoming dialysis-dependent over their lifetime [1, 4].

Due to the rarity of the disease and its slow progression, evaluation of traditional renal endpoints such as kidney failure or doubling of serum creatinine is not realistic in short follow-up periods. In the past, this has discouraged IgAN trial initiators from starting new studies. The identification of surrogate markers, such as proteinuria reduction and the rate of (estimated) glomerular filtration rate (eGFR) decline (eGFR slope), has improved trial feasibility and prompted industry interest in drug development for IgA nephropathy [5–7].

New emerging therapies like targeted-release formulation (Trf) budesonide and sparsentan have recently been approved for the treatment of IgAN, showing convincing effects on proteinuria reduction and disease progression [8]. However, although significantly less frequent than with oral corticosteroids, there is also an increased incidence of adverse events with the new budesonide formulation compared with placebo. Moreover, proteinuria increases again after completion of the treatment, potentially indicating a disease recurrence and the need to continue immunosuppressive therapy for a longer period or even indefinitely [9]. Sparsentan, a dual endothelin and angiotensin II receptor antagonist (DEARA), was approved for the treatment of IgAN in Europe in April 2024 and the USA in September 2024, as the first non-immunosuppressive treatment to improve outcome in IgAN ,with market launch in August 2024 in Germany [10, 11].

Sparsentan exhibited remarkable effects on proteinuria reduction and slowing down IgAN progression in the PROTECT trial (Phase III), particularly in patients with high risk of disease progression (proteinuria >1 g/day) [9, 12] and is now recommended as a treatment option in the public draft of the 2024 KDIGO IgAN/IgAV guideline for IgAN patients with risk of progressive kidney function loss [13]. On the other hand, sodium-glucose co-transporter 2 (SGLT2) inhibitors (SGLTis) have proven efficacy on the progression of CKD in patients with IgAN: in the IgAN subgroup analyses of DAPA-CKD and EMPA-KIDNEY a reduction of CKD progression has been shown [14, 15]. Therefore, in addition to renin–angiotensin system (RAS) inhibition, SGLT2is have already become standard of care for patients with IgAN [8]. However, data on the combined treatment with SGLT2is and sparsentan are lacking, as during the double-blind period of the PROTECT trial SGLT2i initiation was not allowed [12].

Sparsentan was made available in several European countries prior to launch through the Managed Access Programme (MAP) starting in November 2023. Thereby, sparsentan was available for high-risk IgAN patients (as defined by the PROTECT study inclusion criteria) prior to its official approval in Europe and could be used in routine clinical practice.

The aim of this study was to evaluate sparsentan efficacy and tolerability in a real-world setting in patients already treated with existing standard of care, including SGLT2is.

## MATERIALS AND METHODS

### Study design and population

**Figure 1:**
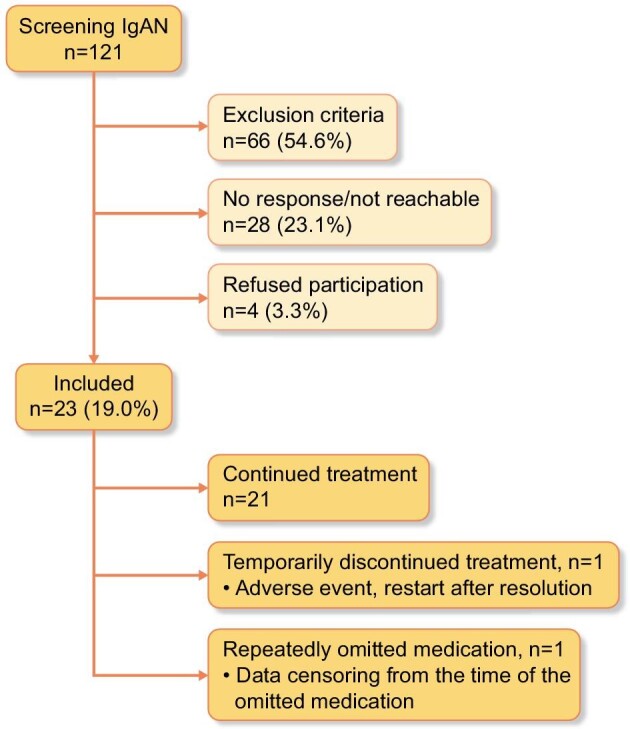
Study flow diagram showing screening and treatment of eligible patients in the context of the MAP in clinical routine (not study-associated).

IgAN patients were treated with sparsentan during clinical routine from November 2023 through the MAP prior to the official approval. In this retrospective multicentre analysis we provide real-world evidence on the efficacy and safety of sparsentan in patients with IgAN during clinical routine (Fig. 1).

The study was conducted in the private practice sector but also outpatient clinics from referral centres across several nephrology centres in Germany: involved centres were the Robert Bosch Hospital in Stuttgart, the Nephrological Center in Villingen-Schwenningen, and the Dialysis Center of the Arbeitsgemeinschaft Heimdialyse Saar e.V. in Homburg.

All patients participating in the MAP with at least one follow-up visit were included in our analysis. The study aimed to include all patients of the MAP, ending by June 2024. The analysis of the enrolled patient data continued until August 2024, prior to the launch of sparsentan in August 2024. Target dose of sparsentan was 400 mg/day after 2 weeks of initial treatment at the half dose according to the MAP study protocol.

Inclusion criteria for our analysis were patients who were on stable SGLT2i therapy (at least >3 months) and participating in the sparsentan MAP. Briefly, inclusion criteria for the MAP were: (i) stable dose of maximal tolerated angiotensin-converting enzyme inhibitor (ACEI) and/or angiotensin receptor blockers (ARBs) for at least 3 months prior to inclusion; (ii) estimated glomerular filtration rate (eGFR) ≥30 mL/min/1.73 m^2^ at eligibility review; (iii) UPCR ≥0.75 g/g or urine protein excretion value ≥1.0 g/day; (iv) mean seated blood pressure of ≥100/60 and ≤160/100 mmHg; (v) aged ≥18 years; (vi) women of childbearing potential had to use effective methods of contraception.

Exclusion criteria were: (i) IgA nephropathy secondary to another condition; (ii) enrolment in an interventional clinical trial; (iii) concomitant use of any of renin-angiotensin-aldosteron system (RAAS)/endothelin inhibitors, strong Cytochrome P450 3A (CYP3A) inhibitors or inducers, potassium-sparing drugs; (iv) undergone organ transplantation; (v) documented heart failure New York Heart Association (NYHA) II–IV or unexplained dyspnoea; (vi) clinically significant cerebrovascular/coronary artery disease within 6 months prior to inclusion; (vii) hepatobiliary disease; (viii) anaemia (haemoglobin <9 g/dL); (ix) potassium values >5.5 mmol/L; (x) hypersensitivity/allergic response to angiotensin receptor blockers (ARB) or endothelin receptor antagonist (ERA); (xi) pregnancy/breastfeeding; (xii) non-adherence in the opinion of the physician.

All patients provided written informed consent for data collection and analysis as part of the MAP. This study was conducted with consultation of the ethics committee of the University of Tuebingen, Germany (384/2024BO2).

### Data collection

Collected data included baseline medication, previous immunosuppressive therapy, and pre-existing conditions. Primary data sources were patient reports, including laboratory values from outpatient care, records with collected data from the MAP, the hospital information system, and the digital archive. No study-associated investigations were conducted; only routine parameters were recorded.

Whenever available, the following data were included: weight, blood pressure, laboratory values, including basic internal laboratory results such as red blood cell (RBC) count, eGFR, glutamic oxaloacetic transaminase (GOT), glutamyl pyruvic transaminase (GPT/ALT), glutamyl pyruvic transaminase (GGT), bilirubin, electrolytes, serum albumin, haematuria, proteinuria, microalbuminuria, kidney biopsy details, such as Oxford classification [16], and interstitial fibrosis and tubular atrophy (IF/TA) grading [17], and all reported adverse events.

### Treatment and follow-up

In the context of MAP, RAS inhibitors and mineralocorticoid receptor antagonists (MRAs) were discontinued 48 hours and 1 week, respectively, before sparsentan initiation.

Follow-up visits were conducted as part of routine examinations at 2, 6, 10, 14, and 22 weeks or as close as possible to these time points. Appointments outside these intervals were aligned with the nearest scheduled follow-up visit.

### Definitions

Treatment response was adapted from the PROTECT study criteria and we only focused on proteinuria (haematuria was not taken into account). As no data on 24-hour urine collection were available [12], response was categorized as follows: partial remission (PR): proteinuria <1.0 g/g, if baseline was between 0.75 and 1.0 g/g, then <0.75 g/g; complete remission (CR): proteinuria <0.3 g/g; no remission (NR) if neither of both criteria were met. If only urine albumin/creatinine ratio (UACR) was available, PR was defined correspondingly with UACR <1000 mg/g and CR with UACR <300 mg/g. Our analysis reports the percentage achieving UPCR or UACR reduction of 30% and 50%, following the approach of the ongoing SPARTACUS trial [18].

Haematuria was analysed automatically as part of the routine measurement (RBCs/microlitre). Haematuria of >28 RBCs/μL was considered positive according to previous work [19]. Obesity was defined according to the World Health Organization (WHO) with a BMI ≥30 kg/m².

### Statistical analysis

For group comparisons of baseline characteristics, categorical variables were analysed using the Fisher exact and *χ*^2^ tests and of continuous variables using the *t*-test and Mann–Whitney test, respectively, for normally and non-normally distributed variables. For paired non-parametric variables the Wilcoxon matched-pairs signed rank test was used. Statistical analysis was performed using Prism (version 10.2.3, GraphPad Software Inc., La Jolla, USA).

## RESULTS

For the MAP a total of *n* = 121 patients with IgAN were screened for eligibility. Of these, 98 patients were excluded due to (i) exclusion criteria (54.6%), (ii) lacking availability/response (23.1%), or (iii) refusal of participation (3.3%) (Fig. 1). The remaining 23 patients participated in the MAP and their clinical data were included in this analysis. The proportions (*n*, %) of patients who completed the corresponding follow-up were: 2 weeks, *n* = 20 (87%); 6 weeks, *n* = 21 (91%); 10 weeks, *n* = 18 (78%); 14 weeks, *n* = 11 (48%); and 22 weeks, *n* = 8 (35%). All patients reached the target dose of 400 mg/day after 2 weeks of initial sparsentan treatment at the half dose according to the study protocol.

### Baseline characteristics

In our cohort, the median age was 38 years (IQR 28–48) and median BMI was 26.8 kg/m² (24.0–30.9) at sparsentan initiation, and 43% were females. The patient group was predominantly White (91%), with Asian patients making up the remaining 9%. Median time from initial kidney biopsy (initial diagnosis of IgAN) to sparsentan initiation was 34 months (10–55) (Table 1A). Most common comorbidities were arterial hypertension (65%), hyperlipoproteinaemia (44%), and obesity (26%). Only a small fraction had diabetes mellitus (9%). Regarding baseline medication, all patients were on stable, maximum tolerated RAS inhibition, and stable SGLT2i therapy. Seventy-four per cent received the maximum allowed RAS inhibition at screening. More than half of the patients (57%) had a history of corticosteroid treatment and most of them had received Trf-budesonide. Four patients (17%) had ongoing budesonide therapy at screening (Table 1A). For detailed breakdown of past (>12 months or <12 months before sparsentan initiation) and ongoing corticosteroid treatment see Supplementary Table S1 and Fig. S1. Further concomitant medication is described in Table 1A.

**Table 1A:** Baseline characteristics at sparsentan initiation.

Patients (*n* = 23)				Median (IQR)
Age at sparsentan initiation (years)				38 (26–48)
Female gender (*n*, %)				10 (43)
Race				
White (*n*, %)				21 (91)
Asian (*n*, %)				2 (9)
Time from initial kidney biopsy to sparsentan initiation (months)				34 (10–55)
BMI (kg/m²)				26.8 (24.0–30.9)
Blood pressure systolic (mmHg)				130 (125–135)
Blood pressure diastolic (mmHg)				80 (76–90)
**Comorbidities**				***N* (%)**

Arterial hypertension				15 (65)
Hyperlipoproteinaemia				10 (44)
Diabetes mellitus				2 (9)
Obesity				6 (26)
**Medication**				***N* (%)**
ACE inhibitors/ARB^a^				23 (100)
ACE inhibitor or ARB at maximum labelled dose at screening				17 (74)
MRA^a^				1 (4)
SGLT2 inhibitors				23 (100)
**History of corticosteroid therapy**				**13 (57)**
	**>12 months**	**<12 months**	**Ongoing**	
Systemic	5 (22)	0 (0)	0 (0)	
TrF-budesonide	1^b^ (4)	3 (13)	4 (17)	
Diuretics				8 (35)
Calcium channel blockers				10 (44)
β-Blockers				3 (13)
Other antihypertensive therapy agents				6 (26)
Lipid-lowering therapy				12 (52)

ACE, angiotensin-converting enzyme; ARB, angiotensin receptor blockers; MRA, mineralocorticoid receptor antagonist; TrF, targeted-release formulation; UPCR, urine protein/creatinine ratio; UACR, urine albumin/creatinine ratio.

a Discontinued prior to sparsentan initiation.

^b^Classic budesonide.

**Table 1B:** Baseline renal parameters at sparsentan initiation.

Baseline renal parameters	Median (IQR)
Baseline eGFR (CKD-EPI) (mL/min/1.73 m²)	42 (32–63)
eGFR decline in the preceding year (mL/min/1.73 m² per year)	
Excluding patients on Trf-budesonide/systemic corticosteroids	−2.8 (−12.0 to 0.5)
Patients on Trf-budesonide/systemic corticosteroids	6.3 (1.9–9.2)
eGFR category (CKD-EPI) (*n*, %)	
≥90 mL/min per 1.73 m²	3 (13)
≥60 to <90 mL/min/1.73 m²	4 (17)
≥45 to <60 mL/min/1.73 m²	4 (17)
≥30 to <45 mL/min/1.73 m²	10 (43)
≥15 to <30 mL/min/1.73 m²^a^	2 (9)
Serum albumin (g/dL)	3.9 (3.7–4.2)
Haematuria (*n*, %)	16 (70)
Baseline UPCR (g/g)	1.5 (0.9–1.8)
Baseline UACR (mg/g)	1254 (786–1665)

TrF, targeted-release formulation.

a eGFR progress <30 mL/min/1.73 m² between screening and sparsentan initiation.

Median baseline eGFR [Chronic Kidney Disease Epidemiology Collaboration (CKD-EPI)] was 42 mL/min/1.73 m² (32–63), whereas distribution across eGFR categories was balanced with a predominance between ≥30 and <45 mL/min/1.73 m² (CKD KDIGO stage G3b) (Table 1B). Median eGFR decline (patients who were on TrF-budesonide/systemic corticosteroids were excluded) in the preceding year before sparsentan initiation was −2.8 mL/min/1.73 m² (−12.0 to 0.5) (Table 1B). Baseline proteinuria (UPCR or UACR) was 1.5 g/g (0.9–1.8) or 1254 mg/g (786–1665), respectively (Table 1B). In our cohort 70% had haematuria (>28 RBCs/μL).

### Histological characteristics/Oxford MEST-C scoring

Regarding histological characteristics, there was a predominance of patients who had evidence of mesangial hypercellularity (M) (72%) and only a small proportion of endocapillary hypercellularity (E) (11%), whereas most of the patients had segmental sclerosis (S) (89%) and absent (56%) or mild (33%) interstitial fibrosis/tubular atrophy (T). Only four (22%) patients had evidence of crescents (C) (Table 2). Concerning general histological changes, most patients had mild (38%) or moderate (43%) global glomerulosclerosis (Table 2).

**Table 2:** Histological characteristics/Oxford MEST-C scoring system (*n*, %).

Score	M (0, 1)	E (0, 1)	S (0, 1)	T (0, 1, 2)	C (0, 1, 2)	IF/TA category	fsGS category	gGS category
0	5 (28)	16 (89)	2 (11)	10 (56)	14 (78)	3 (13)	3 (15)	2 (10)
1	13 (72)	2 (11)	16 (89)	6 (33)	4 (22)	12 (52)	12 (60)	8 (38)
2				2 (11)	0 (0)	6 (26)	3 (15)	9 (43)
3						2 (9)	2 (10)	2 (10)
Not stated/possible	5 (22)	5 (22)	5 (22)	5 (22)	5 (22)	0 (0)	3 (13)	2 (7)

MEST-C scores: M, mesangial hypercellularity; E, endocapillary hypercellularity; fsGS, focal-segmental glomerulosclerosis; gGS, global glomerulosclerosis; S, segmental sclerosis; T, interstitial fibrosis/tubular atrophy; C, crescents, IF/TA, interstitial fibrosis and tubular atrophy.

IFTA/fsGS/gGS categories: 0, absent; 1 (mild), <25%; 2 (moderate), 25–50%; 3 (severe), >50% of total area or of the glomeruli, respectively.

### Proteinuria reduction

**Figure 2:**
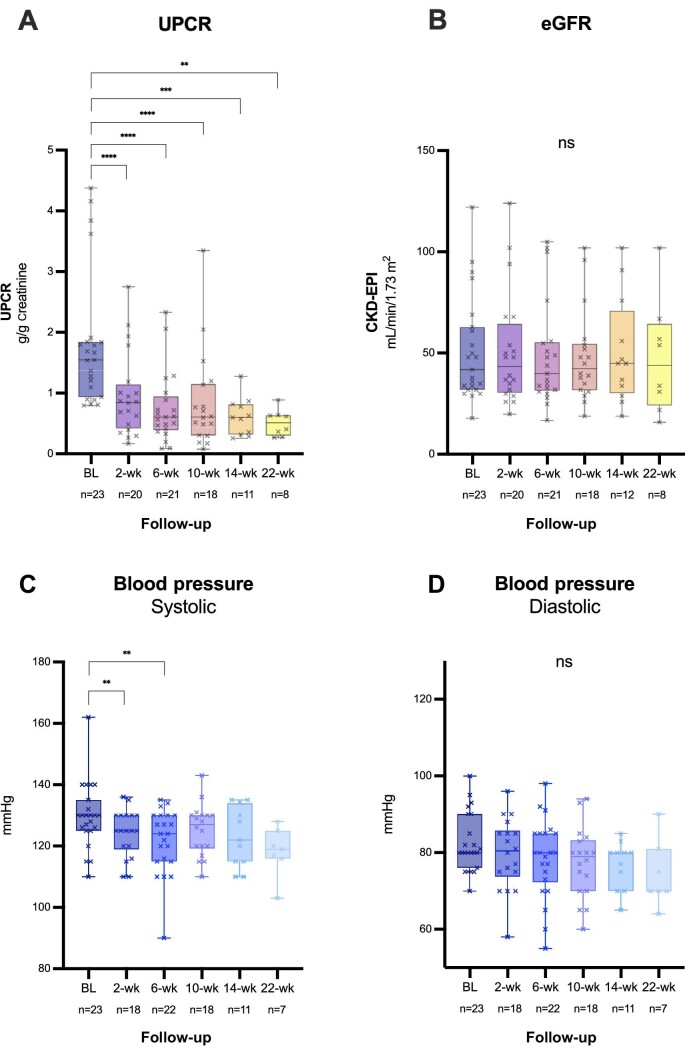
Course of proteinuria (UPCR) (**A**), eGFR (**B**), systolic (**C**) and diastolic (**D**) blood pressure under therapy with sparsentan. Box, median, IQR; whisker, minimum–maximum. BL, baseline; CKD-EPI, Chronic Kidney Disease Epidemiology Collaboration; ns, not significant; wk, week.

In our cohort, significant reductions of UPCR across the follow-ups were shown: (i) 2 weeks (*P* < 0.0001), median −0.85 g/g (0.42–1.15), corresponding to a relative reduction of −44% (17–68); (ii) 6 weeks (<0.0001), −0.61 g/g (0.40–0.95), corresponding to 62% (54–77); (iii) 10 weeks (<0.0001), −0.61 g/g (0.31–1.16)/−53% (34–68); (iv) 14 weeks (*P* = 0.001), −0.60 g/g (0.32–0.82)/−62% (45–74); and (v) 22 weeks (*P* = 0.008), −0.52 g/g (0.30–0.64)/−65% (56–77) (Fig. 2A and Table 3). Similar results were seen for UACR (Table 3). The significant proteinuria reduction under therapy with sparsentan persisted even after excluding patients (i) with ongoing corticosteroid therapy or (ii) with ongoing AND past (<12 months) corticosteroid therapy (Fig. S2).

**Table 3:** Course of proteinuria (UPCR/UACR) under therapy with sparsentan.

	UPCR (g/g)	Reduction (%)			UACR (mg/g)	Reduction (%)		
	Median (IQR)		*n*	*P*-value^a^	Median (IQR)		*n*	*P*-value^a^
Baseline	1.55 (0.94–1.85)	Reference	23		1254 (786–1665)	Reference	23	
2-week follow-up	0.85 (0.42–1.15)	44 (17–68)	20	<0.0001	710 (333–1134)	39 (20–60)	17	0.0002
6-week follow-up	0.61 (0.40–0.95)	62 (54–77)	21	<0.0001	489 (302–699)	65 (50–73)	18	0.0002
10-week follow-up	0.61 (0.31–1.16)	53 (34–68)	18	<0.0001	459 (213–1034)	51 (26–69)	16	<0.0001
14-week follow-up	0.60 (0.32–0.82)	62 (45–74)	11	0.001	490 (288–732)	60 (41–77)	11	0.001
22-week follow-up	0.52 (0.30–0.64)	65 (56–77)	8	0.008	364 (181–562)	68 (62–81)	8	0.008

a Between baseline and follow-up (absolute results); Wilcoxon matched-pairs signed rank test.

**Figure 3:**
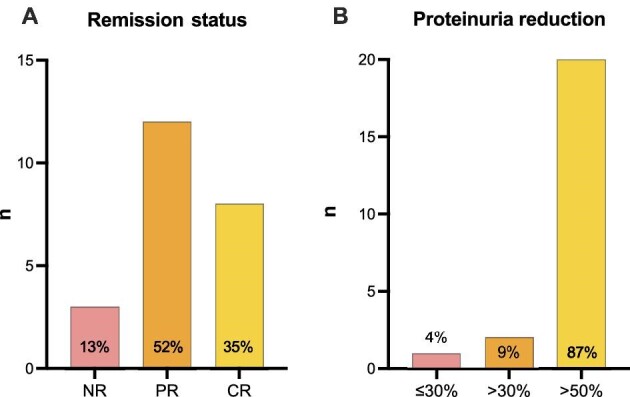
(**A**) Proportion of patients reaching complete/partial remission of proteinuria. Definition of proteinuria at least once at any time over the course of the treatment period: complete remission (CR) UPCR <0.3 g/g; partial remission (PR) is defined as <1.0 g/g, if baseline was between 0.75 and 1.0 g/g, then <0.75 g/g. (**B**) Proportion of patients with >30% or >50% UPCR reduction at any time during treatment in the observation period.

Regarding remission status, CR was achieved in 35% and PR was evident in 52% of the patients (Fig. 3A). Comparable results were recorded for UACR results.

The proportion with UPCR reduction of >30% or >50% was 9% or 87% respectively (Fig. 3B).

### Time course of variables

The laboratory values remained stable, showing no significant changes over time (Table 4). In particular, eGFR also stayed stable (Fig. 2B). Blood pressure values showed a significant trend towards a slight decrease at weeks 2 and 6 (*P* = 0.006 and *P* = 0.003) followed by an incline towards initial levels at week 10 (Fig. 2C and D). Body weight did not change significantly (*P* > 0.05) over time under sparsentan treatment (Table 4).

**Table 4:** Clinical and laboratory results during sparsentan treatment.

	Baseline	2-week	6-week	10-week	14-week	22-week
Clinical variables, median (IQR)						
Weight (kg)	80 (70–92)	81 (73–91)	81 (71–88)	80 (71–87)	82 (74–93)	81 (72–92)
Blood pressure systolic (mmHg)	130 (125–135)	125 (119–130)	124 (115–130)	127 (119–130)	122 (115–134)	119 (116–125)
Blood pressure diastolic (mmHg)	80 (76–90)	81 (74–86)	80 (72–85)	79 (70–83)	80 (70–80)	70 (70–81)
Laboratory variables, median (IQR)						
eGFR (CKD-EPI) (mL/min/1.73 m^2^)	42 (32–63)	44 (31–65)	40 (32–56)	43 (32–55)	45 (30–71)	44 (24–65)
Serum creatinine (mg/dL)	1.8 (1.3–2.3)	1.7 (1.1–2.3)	1.9 (1.4–2.4)	1.8 (1.5–2.3)	1.8 (1.2–2.5)	1.6 (1.1–2.6)
Urea (mg/dL)	56 (38–79)	56 (41–84)	60 (42–90)	69 (45–82)	71 (40–103)	63 (36–91)
NT-proBNP (pg/mL)	54 (22–89)	65 (28–116)	56 (32–158)	75 (34–126)	89 (27–179)	90 (47–270)
Bilirubin (mg/dL)	0.4 (0.3–0.7)	0.4 (0.3–0.6)	0.4 (0.3–0.5)	0.4 (0.3–0.5)	0.4 (0.3–0.5)	0.5 (0.3–0.6)
GOT/AST (U/L)	18 (14–25)	21 (15–29)	20 (17–24)	22 (16–27)	20 (14–35)	23 (13–44)
GPT/ALT (U/L)	22 (14–31)	22 (12–35)	21 (13–26)	21 (13–24)	22 (15–34)	23 (20–49)
GGT (U/L)	23 (15–27)	21 (15–26)	22 (15–27)	19 (14–30)	24 (15–34)	26 (14–43)
Leucocytes (10^9^/L)	7.9 (6.7–9.2)	7.3 (6.3–8.7)	6.6 (5.8–9.5)	6.9 (5.5–8.4)	6.7 (5.5–8.6)	6.3 (5.7–8.2)
Haemoglobin (g/L)	143 (132–156)	137 (129–155)	136 (130–148)	132 (127–147)	140 (126–149)	128 (123–150)
Platelets (10^9^/L)	264 (217–306)	257 (217–290)	251 (223–277)	235 (208–261)	216 (183–256)	175 (162–230)
Bicarbonate (mmol/L)	24 (22–25)	22 (21–23)	22 (21–24)	22 (20–23)	21 (21–23)	20 (19–22)
Potassium (mmol/L)	4.2 (3.9–4.5)	4.2 (4.0–4.7)	4.3 (4.0–4.6)	4.4 (4.2–4.7)	4.4 (4.3–4.6)	4.4 (4.2–4.9)

NT-proBNP, N-terminal pro-hormone B-type natriuretic peptide.

### Safety

Sparsentan was well tolerated with no evidence of drug-related serious adverse events. Only a few mild adverse events were recorded within the context of the MAP that were likely associated with sparsentan treatment (Supplementary Table S1): two events (8.7%) of hypotension, dizziness (*n* = 1; 4.3%), oedema (*n* = 1; 4.3%), headache (*n* = 1; 4.3%), mild hyperkalaemia (definition ≤5.5 mmol/L) (*n* = 1; 4.3%), and pruritus (*n* = 1; 4.3%). One patient temporarily discontinued treatment due to symptomatic hypotension for 4 weeks (because of the Christmas holidays); after restarting, the symptoms no longer occurred, even after titration to the target dose. No patient had to permanently discontinue sparsentan treatment. Adverse events that were unlikely to be related to sparsentan treatment were gout (*n* = 1; 4.3%) and hospital admission for pneumonia (serious adverse event) (*n* = 1; 4.3%) (Supplementary Table S1).

## DISCUSSION

To our knowledge, this is the first real-world analysis assessing sparsentan in the treatment of IgAN alongside stable supportive care, including SGLT2is.

In our analysis, we were able to demonstrate a significant decrease of UPCR and UACR under sparsentan treatment comparable to effects observed in the PROTECT trial. Importantly, also patients who were already receiving stable SGLT2 inhibition and maximally tolerated/allowed RAS inhibition before initiation of sparsentan showed a sustained and further improving effect on proteinuria reduction. We observed a significant relative UPCR or UACR reduction of >60% (weeks 6, 14, and 22). In our cohort 9% achieved >30% and 87% achieved >50% UPCR reduction, meaning that only 4% displayed a small UPCR reduction of ≤30%. Complete proteinuria remission rate was 35%, which is higher than in the PROTECT collective (21%) [12].

Preliminary data from the open-label extension trial of the PROTECT study, presented in abstract form at the World Congress of Nephrology in April 2024, where SGLT2i therapy could be added at the investigator's discretion to ongoing sparsentan therapy after the double-blind study period, are consistent with our data: a median UPCR reduction up to 44% (week 36; *n* = 16) was achieved, confirming our findings on the add-on effect [20], which is also discussed in other drugs combined with SGLT2is, like semaglutide [21]. However, these data only originate from a selected study collective and do not reflect the real-world situation. Moreover, the addition of SGLT2i therapy to ongoing sparsentan treatment is an unusual sequence in routine clinical practice [20].

In IgAN, as a rare and mostly slowly progressive disease, evaluation of traditional renal endpoints is not feasible in short follow-up periods such as ours. Even in the context of large studies such as the PROTECT study, primary surrogate endpoints such as proteinuria reduction or eGFR slopes were evaluated, which are accepted by the FDA/EMA as such surrogate endpoints [5–7]. In our collective, the time period was still too short for eGFR-slope analyses, and therefore only the proteinuria reduction was analysed. New data from the German CKD (GCKD) cohort reaffirm proteinuria/albuminuria to be the strongest predictor of poor kidney outcome in IgAN and emphasize the importance of targeting UACR reduction in clinical management of IgAN to improve long-term kidney outcome [4]. Nevertheless, the eGFR time course was largely stable over time.

The responsible effects of sparsentan that contribute to proteinuria reduction have not yet been fully clarified. Haemodynamic effects are being discussed [22] alongside direct effects on mesangial cells and podocytes. In principle, a proportion certainly seems to be haemodynamically induced, as a slight systolic blood pressure dip (baseline visit to first and second follow-ups after 2 and 6 weeks, respectively) was generally recorded after the start of therapy and proteinuria showed a marked decrease. However, we also saw that even after stabilization of blood pressure and a partial increase in blood pressure values, proteinuria was persistently low, so that the effect cannot be explained by haemodynamics alone and other effects must certainly be responsible for the reduction in proteinuria. Recently, Nagasawa *et al*. demonstrated in the gddY mouse model of IgAN a prevention of glycocalyx and podocyte damage and glomerulosclerosis through sparsentan despite a comparable antihypertensive effect, affirming the assumption of an additional podocyte-stabilizing effect beyond haemodynamics alone [23].

There are also other selective endothelin receptor A antagonists emerging for IgAN treatment: interim results from the Phase 3 ALIGN trial showed that atrasentan significantly reduced proteinuria by 38.1% at week 36 compared with 3% in the placebo group, highlighting this class of drugs as a promising therapeutic approach for IgAN [24].

Concerning the safety profile of sparsentan, we were able to confirm the safety and tolerability in our real-world setting as described in PROTECT: sparsentan therapy is non-toxic and well tolerated, and the adverse side effects in our MAP were even lower than in the pivotal study, which is probably due to the shorter observation time. The adverse events were generally minor and only mild. There were no sparsentan related serious adverse events. As in the PROTECT study, the most common adverse events were dizziness, hypotension, and headache, with an incidence in our analysis of <10% [12]. There was no permanent discontinuation of therapy in any patient. Only one patient (first patient) who was symptomatically hypotensive paused treatment for a short time, as there was only limited experience at this time. In particular, there were no relevant signals regarding fluid retention. Using the ETA receptor antagonist atrasentan, the SONAR trial showed a higher rate of fluid retention in patients with diabetic nephropathy in the atrasentan arm [18, 25]. In our collective we could not detect such signals, as the weights did not differ significantly over the treatment period. A possible explanation could either be substance-specific characteristics or the protective effect of SGLT2 inhibition, which has a certain diuretic effect and thus counteracts fluid retention [26]. Additional, very recent evidence of the long-term efficacy and safety of sparsentan is available from the open-label extension of the DUET trial in patients with focal segmental glomerulosclerosis over a treatment period of >4 years, where long-term sparsentan therapy showed sustained proteinuria reduction and a consistent safety profile [27].

However, our study has of course several limitations: this was a real-world setting, i.e. it was not performed in a controlled study environment with rigorous monitoring and follow-up. Some patients were treated in an outpatient setting by nephrologists in private practice, which limited the availability of follow-up data and made it difficult to closely reproduce the course of treatment during analysis for the study team. In addition, vital signs and laboratory values tend to be more incomplete in a real-world setting than in a controlled study setup.

On the other hand, our analysis has also unique strengths. Our collective is fairly representative of all IgAN patients. (i) We have a higher rate of female participants, with 43% compared with PROTECT (31%). (ii) Regarding patients with a history of corticosteroid therapy, ∼57% had a history or ongoing corticosteroid therapy, which is a typical medical history of high-risk IgAN patients. (iii) Most of them had targeted-release budesonide, a newly emerging treatment option, so that this new drug is already included in our collective, and we could demonstrate that even after exclusion of corticosteroid (mostly TrF-budesonide)-treated patients—which may have possibly biased the results—the significant effect of sparsentan on UPCR reduction persisted. (iv) Our analysis was a multicentre study from all areas: not only the private practice sector but also also outpatient clinics from referral centres, which significantly increases the generalizability.

We therefore consider the applicability or external validity to be significantly higher compared with the previous study settings.

## CONCLUSIONS

In conclusion, in a real-world setting sparsentan demonstrates a significant and sustained add-on effect of existing supportive therapy including SGLT2is on proteinuria and albuminuria, resulting in a relative reduction of 62% (45–74) in UPCR after 14 weeks and beyond, demonstrating great potential for improving IgAN prognosis.

## Supplementary Material

sfae394_Supplemental_Files

## Data Availability

The data underlying this article will be shared on reasonable request to the corresponding author after internal board review.
