# Supplementary material for: First real-world evidence of sparsentan efficacy in patients with IgA nephropathy treated with SGLT2 inhibitors
Source: Clin Kidney J. 2024 Dec 3;18(1):sfae394. doi: 10.1093/ckj/sfae394 (PMC11770278; doi:10.1093/ckj/sfae394)
Supplement: sfae394_Supplemental_Files [file sfae394_supplemental_files.zip › 970 supplementary material.docx]

**Table S1.** Detailed breakdown of past (longer than 12 months or in the past 12 months before sparsentan initiation) and ongoing corticosteroid treatment: Specification of quantity, time between corticosteroid discontinuation and sparsentan initiation and duration of therapy depending on the type of treatment (systemic or TrF-budesonide).

|  | >12 months | | | <12 months | ongoing |
| --- | --- | --- | --- | --- | --- |
| History of corticosteroid therapy, n (%) |  | | |  |  |
| Systemic | 5 (22) | | | 0 (0) | 0 (0) |
| TrF-budesonide | 1^+^ (4) | | | 3 (13) | 4 (17) |
|  |  | | |  |  |
| Time between corticosteroid termination and sparsentan initiation (m) Median (IQR) | |  | |  |  |
| Systemic | 47 (23-81) | | | - | - |
| TrF-budesonide | 30 | | | 3 (2-4) | -1 (-3- -1) |
|  |  | | |  |  |
| Therapy duration (m), Median (IQR) |  | | |  |  |
| Systemic | 6 (6-22) | | | - | - |
| TrF-budesonide | 9 | | | 7 (6-9) | 9 (8-9) |
|  |  | |  | |  |

Abbreviations: IQR: Interquartile range; TrF: Targeted-release formulation; m: month; wk: Week.

^+^Classic budesonide.

**Table S2.** Adverse events possibly related and unlikely related to sparsentan treatment.

| Event | n (%) |
| --- | --- |
| Possibly related to sparsentan treatment |  |
| Hypotension | 2 (8.7) |
| Dizziness | 1 (4.3) |
| Oedema | 1 (4.3) |
| Mild hyperkalaemia (≤5.5 mmol/L) | 1 (4.3) |
| Headache | 1 (4.3) |
| Pruritus | 1 (4.3) |
|  |  |
| Unlikely related to sparsentan treatment |  |
| Gout | 1 (4.3) |
| Hospitalisation for pneumonia (SAE) | 1 (4.3) |

Abbreviations: SAE: Serious adverse event.

**Figure S1.** Overview of corticosteroid therapy (all patients received TrF-budesonide) in the year before (<12 months) and during (ongoing) the start of sparsentan.

Abbreviations: TrF: Targeted-release formulation.

**Figure S2.** Course of proteinuria (UPCR) under therapy with sparsentan: (B) excluding patients with ongoing corticosteroid therapy, and (C) with ongoing corticosteroid therapy AND in the year before (<12 months) compared to the entire cohort (A). Box: Median, IQR; Whisker: Min-Max.

Abbreviations: BL: Baseline; eGFR: estimated glomerular filtration rate; IQR: Interquartile range; TrF: Targeted-release formulation; UPCR: Urine protein-creatinine ratio; wk: Week.

**Figure S1**

**
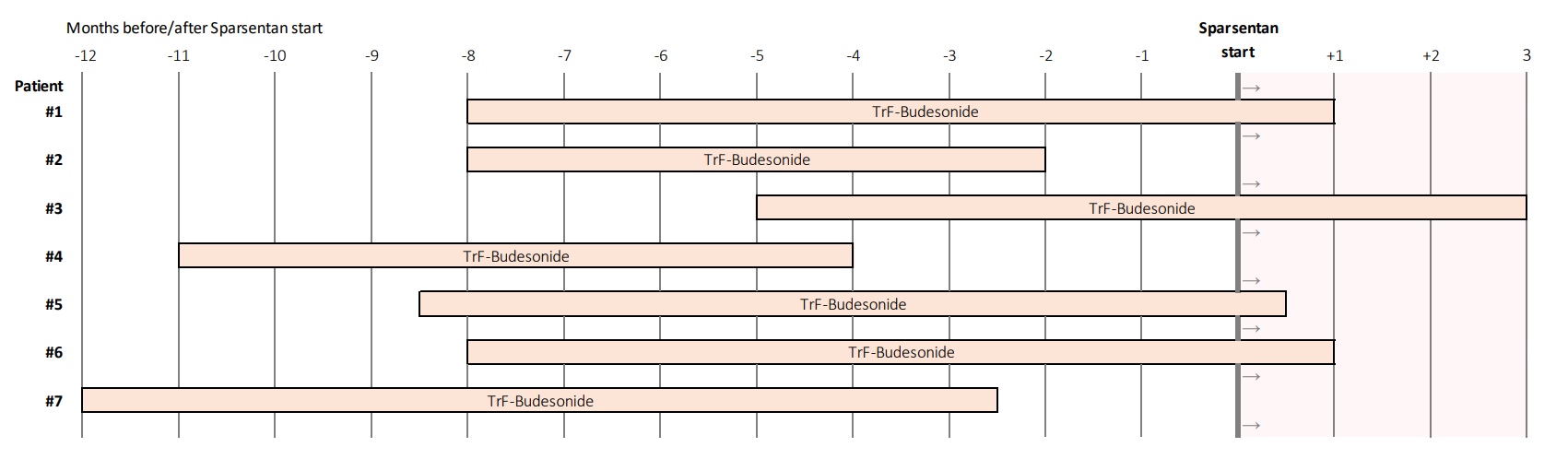
**

**Figure S2**

**
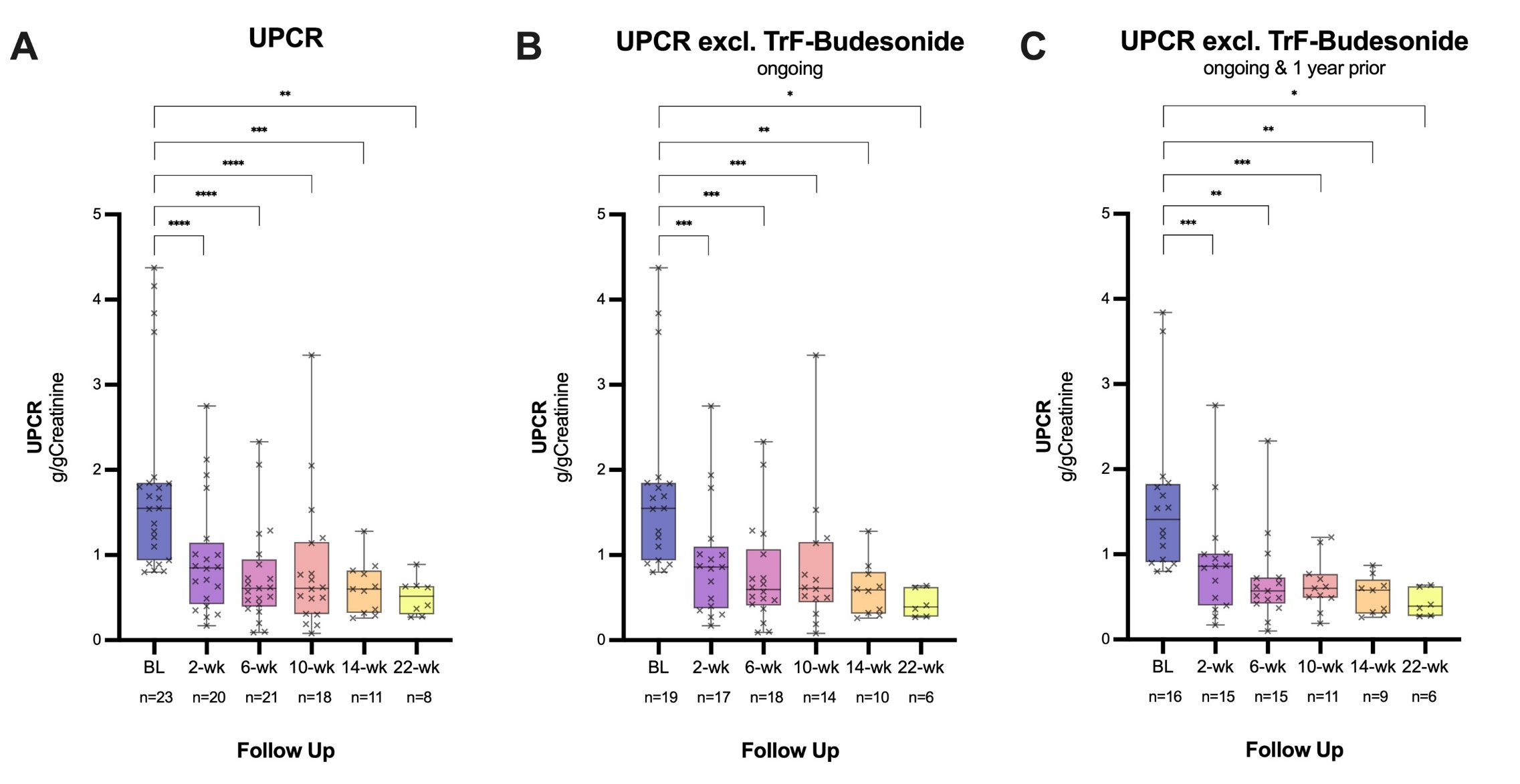
**
