# Supplementary figures and images for: First real-world evidence of sparsentan efficacy in patients with IgA nephropathy treated with SGLT2 inhibitors
Source: Clin Kidney J. 2024 Dec 3;18(1):sfae394. doi: 10.1093/ckj/sfae394 (PMC11770278; doi:10.1093/ckj/sfae394)

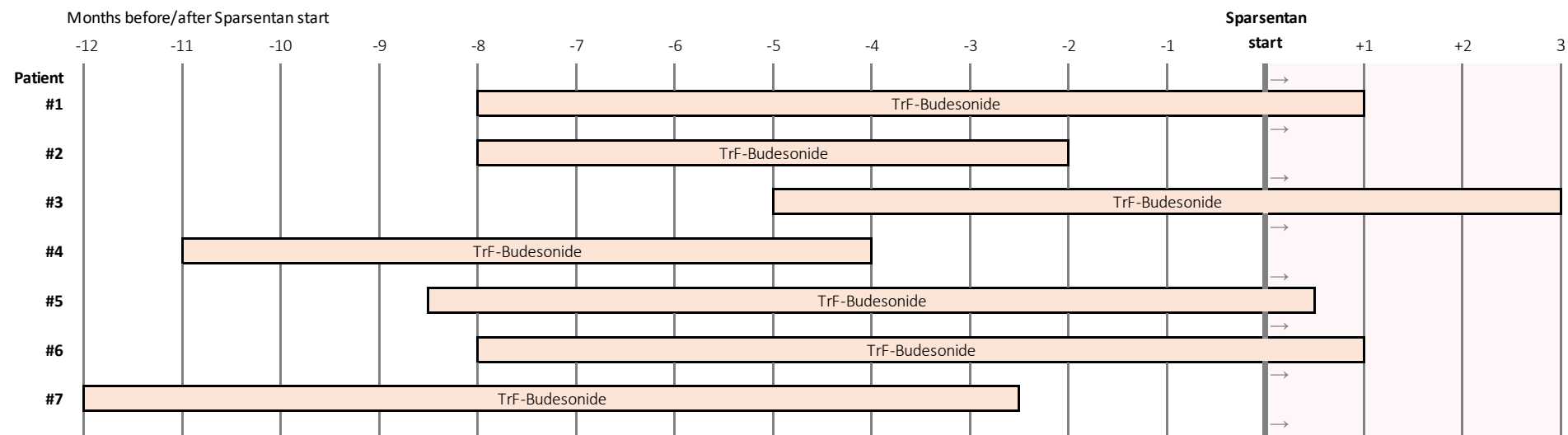

Supplement: sfae394_Supplemental_Files [file sfae394_supplemental_files.zip › FigureS1_R1.pdf]
